# So much to teach, so little time: a prospective cohort study evaluating a tool to select content for a critical care curriculum

**DOI:** 10.1186/cc7087

**Published:** 2008-10-15

**Authors:** Adam D Peets, Kevin McLaughlin, Jocelyn Lockyer, Tyrone Donnon

**Affiliations:** 1 Department of Critical Care Medicine, University of Calgary, 29th St NW, Calgary T2N 2T9 Canada; 2 Department of Medicine, University of Calgary, Hospital Drive NW, Calgary T2N 4N1, Canada; 3 Department of Community Health Sciences, University of Calgary, Hospital Drive NW, Calgary T2N 4N1, Canada

## Abstract

**Introduction:**

Curricular content is often based on the personal opinions of a small number of individuals. Although convenient, such curricula may not meet the needs of the target learner, the program or the institution. Using an objective method to ensure content validity of a curriculum can alleviate this issue.

**Methods:**

A form was created that listed clinical presentations relevant to residents completing intensive care unit (ICU) rotations. Twenty residents and 20 intensivists in tertiary academic multisystem ICUs ranked each presentation on three separate scales: how life-threatening each is, how commonly each is seen in critical care, and how reversible each is. Mean scores for the individual scales were calculated, and these three values were subsequently multiplied together to achieve a composite score for each presentation. The correlation between the two groups' scores for the presentations was calculated to assess reliability of the process.

**Results:**

There was excellent agreement between the two groups for rating each presentation (correlation coefficient *r *= 0.94). The 10 clinical presentations with the highest composite scores formed the basis of our new curriculum.

**Conclusions:**

We describe a method that can be used to select the content of a curriculum for learners in an ICU. Although the content that we selected to include in our curriculum may not be applicable to other ICUs, we believe that the process we used is easily applied elsewhere, and that it provides an efficient method to improve content validity of a curriculum.

## Introduction

Learning within the intensive care unit (ICU) environment is extremely challenging, not only because of the rapid pace of patient care but also because of the depth and breadth of knowledge required to care for critically ill patients. Large scale projects around the world have been undertaken to define encompassing knowledge objectives for learners in the critical care setting [1-5]. However, given the time constraints associated with clinical practice, it is not possible to teach learners about every topic related to critical care medicine. So how should we select the most relevant content to include in a curriculum for trainees with a limited amount of time in the ICU?

One method to standardize this process is to begin by obtaining input from key stakeholders [6-8]. If this is done in an objective and systematic manner, it can result in improved relevance of the content and can facilitate the implementation of the new curriculum [9].

As part of the process of curricular revision at our institution we developed a tool, which we then used to identify appropriate content for our curriculum. Our objective here is to describe the process that we undertook, which we believe can be adopted by others involved in curriculum development.

## Materials and methods

The Department of Critical Care Medicine in the Calgary Health Region consists of three adult academic ICUs. Each is a multidisciplinary medical/surgical unit staffed by board certified intensivists. Residents from 17 programmes complete rotations between 4 and 12 weeks in length. Based on resident and attending physician feedback, a decision was made to revise the existing educational curriculum.

A technique similar to that employed by McLaughlin and coworkers [10] was used for the needs assessment. They asked clinical clerks and specialists in general internal medicine to rate 47 clinical presentations relevant to an internal medicine clerkship rotation on two scales: impact and frequency. For each clinical presentation the mean scores on each scale were multiplied, resulting in a score that was used to evaluate relevance of material to their new curriculum. In order to make the form more relevant to the ICU setting, we replaced the 'impact' scale with two scales: how life-threatening a clinical presentation is and how reversible it is. A comprehensive list of clinical presentations that are potentially relevant to residents completing rotations in an ICU was created using information from the Society of Critical Care Medicine [3], the Royal College of Physicians and Surgeons of Canada [4] and the Medical Council of Canada [11]. These presentations were subsequently listed in alphabetical order on a sheet and distributed to key stakeholders.

We identified residents and critical care medicine attending physicians as our key stakeholders. For each of the presentations we asked participants to assign a numerical value from 1 to 3 based on the descriptions given in Table 1 for each of three scales: life-threatening (how quickly it results in death), frequency (how commonly it is seen in the ICU) and reversibility (how reversible it is with treatment). Therefore, a presentation could be considered important if it is very common, rapidly life-threatening and easily reversible. Before distribution, 10 postgraduate year 5 residents, two experts in critical care medicine and three experts in medical education, reviewed the tool to optimize face and content validity.

**Table 1:** Needs assessment scoring system

**Clinical Presentation**	**Life-threatening**	**Frequency**	**Reversibility**
	1 = Within days	1 = Rarely seen	1 = Not reversible
	2 = Within hours	2 = Relatively common	2 = Potentially reversible
	3 = Within minutes	3 = Very common	3 = Easily reversible

Acid-base disorders			

Acute abdomen			

Acute pancreatitis			

*Continued list of clinical presentations...*			

We calculated the mean scores for the scales of life-threatening, frequency and reversibility for each of the 37 clinical presentations rated by each group. Initially, the mean scores were multiplied together to create a composite score that could range between 1 and 27, and then the procedure was repeated by adding them together to create a separate composite score that could range from 3 to 9. For each of the clinical presentations, we calculated a mean value for the two composite scores from the residents and attendings to produce a final score. Presentations were ranked based on this final score. Because the curriculum would be delivered over a 1-month period, we decided *a priori *that the top 10 clinical presentations would form the basis of the new curriculum.

To determine whether multiplication or addition of the scales provided more robust results, we used the same technique as that used by McLaughlin and coworkers [10]. Simple regression analysis using the residents' mean scores as the independent variable and attendings' mean scores as the dependent variable was completed for multiplication initially and then repeated for addition. This process was undertaken to assess the goodness-of-fit for each model, as reflected by the *R*^2 ^value. We also calculated the correlation between the mean scores of faculty and residents using Pearson's correlation coefficient to assess the inter-rater reliability of our process. Finally, we created boxplots using the final scores for the two methods with 95% confidence intervals to assess the face validity of the two techniques.

Because completion of the tool was voluntary, participants were considered to have given consent if it was completed and returned. The Conjoint Health Research Ethics Board for the University of Calgary and Calgary Health Region approved this study before its initiation.

## Results

Between January and February 2006, all 20 critical care medicine attending physicians in the Calgary Health Region completed the tool. We randomly sampled 40 of the 276 residents who had completed ICU rotations within the past 2 years. We invited these residents to participate through e-mail notification and 20 completed the exercise. Nine women and 11 men from the following training programs replied: internal medicine (n = 4), surgery (n = 2), anaesthesia (n = 4), emergency medicine (n = 3), family medicine (n = 4), neurology (n = 2) and pathology (n = 1).

The final list of clinical presentations, ranked by composite score, is presented in Table 2. The rank order of the top ten presentations was identical whether multiplication or addition of the scales was used. Overall, there was excellent agreement between the rankings of attendings and residents, with a Pearson correlation coefficient *r *of 0.94. The agreement between the groups on each of the scales was also excellent, with correlation coefficients of 0.91, 0.93 and 0.89 for the scales of life-threatening, frequency and reversibility, respectively. The content of the new curriculum consisted of acute respiratory failure, cardiac dysrhythmias, shock, derangements of electrolytes, acid-base derangements, seizure, cardiac arrest, drug overdose and withdrawal, multisystem trauma and sepsis.

**Table 2:** Mean scores for each clinical presentation rated by residents and attending physicians

Clinical presentations	Resident	Attending
1. Acute respiratory failure	18.0	18.0

2. Cardiac dysrhythmia	15.6	18.4

3. Shock	17.3	15.0

4. Derangements of electrolytes and osmolality	15.5	14.7

5. Acid-base derangements	14.0	14.8

6. Seizure	14.4	13.6

7. Cardiac arrest	13.5	13.8

8. Drug overdose and withdrawal	13.6	12.4

9. Multisystem trauma	13.2	12.7

10. Sepsis	12.9	11.2

11. Diabetes mellitus and its complications	11.1	12.9

12. Chest pain	11.9	11.4

13. Gastrointestinal bleeding	11.9	10.9

14. Acute defects in haemostasis	11.8	10.2

15. Coma	9.3	11.3

16. Raised intracranial pressure	9.0	10.5

17. Perioperative management	10.0	8.3

18. Hypertensive emergency	8.4	9.7

19. Acute abdomen	9.3	8.6

20. The febrile ICU patient	9.6	7.2

21. Traumatic brain injury	7.2	9.4

22. Nontraumatic intracranial bleed	8.7	7.5

23. Delirium	8.7	7.2

24. Acute renal failure	8.0	7.8

25. Adrenal crisis	5.4	7.0

26. Obstetrical complications	5.7	6.4

27. End-of-life decision-making	4.7	7.6

28. Burns	5.4	6.4

29. Rhabdomyolysis	6.2	5.5

30. Diarrhoea in the ICU patient	5.8	5.8

31. Anoxic brain injury	4.4	6.7

32. Near drowning	5.0	5.2

33. Polyuria	4.6	5.1

34. Acute pancreatitis	4.4	5.3

35. Acute neuromuscular disease	5.2	4.1

36. Acute and fulminant hepatic failure	3.2	4.2

37. Patient with brain death	2.6	3.6

The mean scores for each clinical presentation were obtained by multiplying scores for each category of life-threatening, frequency and reversibility together. Rank order was determined by averaging resident and attending mean scores for each clinical presentation. ICU, intensive care unit.

**Figure 1:**
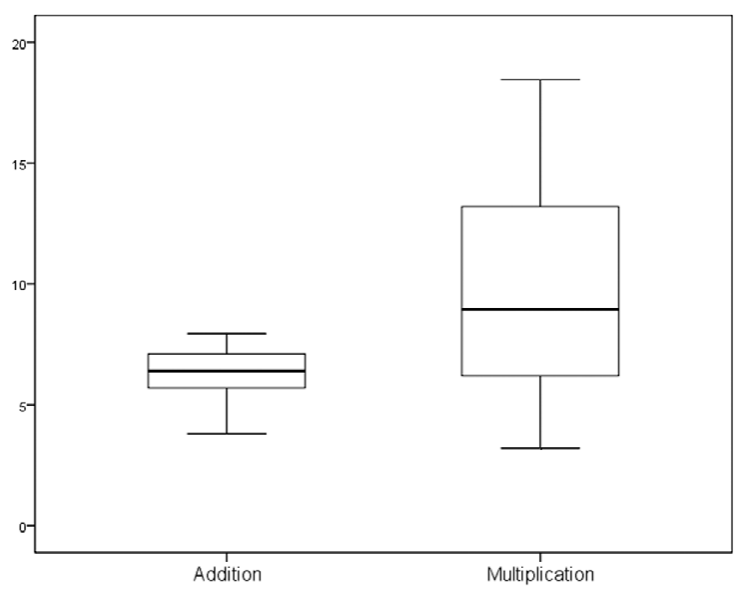
Distribution of composite scores for clinical presentations based on either addition or multiplication of scales. The scales were as follows: life-threatening, frequency and reversibility. Boxplot lines reflect minimum, first quartile, median, third quartile, and maximum moving inferiorly to superiorly.

The results of the regression analysis revealed an *R*^2 ^of 0.87 for the technique of multiplying the scales together, as compared with an *R*^2 ^of 0.78 for the additive technique. Figure 1 demonstrates that the technique of multiplication results in a more positive skew toward the clinical presentations considered to be more life-threatening, more reversible and encountered more frequently, whereas adding the scores together results in a positive skew toward what could be considered by this tool as less important presentations.

## Discussion

In this study we have described both the tool and method used to determine the content for our critical care curriculum for rotating residents. The content chosen – the 10 highest ranked clinical presentations – is valid because it reflects the type of patients seen in our ICUs and the opinions of both learners and preceptors.

The selection of curricular content is often based upon the personal opinions of a small number of educators involved in their development. Although convenient, this process may produce a curriculum that fails to meet the needs of the target learner, the program or the institution. The tool used in this study offers an efficient way of quantifying what key stakeholders believe is the most appropriate content for a critical care rotation. This technique can easily be adapted to any level of learner, in any programme, within any institution, and it provides a strong foundation for the content validity of the curriculum.

To our knowledge, this is the first time that a model using three scales has been used to assess the relative importance of a clinical presentation. For the purposes of this study, it was felt that the 'impact scale' used by McLaughlin and coworkers [10] would not provide adequate discrimination between clinical presentations because the majority of them seen within an ICU could be considered high impact. Therefore, the frequency scale was retained and the new scales of reversibility and life-threatening were added. During the piloting of the questionnaire both residents and attending physicians commented that the three scales were relevant and easily applied to the great majority of the clinical presentations that were listed.

Whether the scales were multiplied or added together to create the composite scores made no difference in the final ranking of the top 10 clinical presentations. Our decision to use the multiplication technique in the final analysis was based upon the positive skew toward the most common, treatable and life-threatening conditions seen by intensivists in our centre and the higher *R*^2 ^in regression analysis. Similar to McLaughlin and coworkers [10], we believe that either technique could be used, but that in this case greater face validity is provided by the multiplication technique.

Upon reviewing the final list of the top 10 clinical presentations, each is extremely important within critical care medicine. However, there are other presentations such as acute renal failure, acute gastrointestinal bleeding and delirium that some may consider equally or even more important. The method that was used in this study to rank the presentations allows for this decision to be undertaken using a quantitative and objective tool, and it therefore maximizes accountability for the content of the final curriculum. Despite residents only having limited clinical experience in the critical care setting, our results demonstrate excellent agreement between the two groups, suggesting consensus. This degree of agreement is also in keeping with previous research in this area [10].

There are some limitations to this process. Our method of scoring may not have been appropriate for some topics. For example, end-of-life decision making, although very relevant to the critical care environment, was not rated highly because low scores for reversibility and how life-threatening it is. Therefore, some topics may need to be considered separately to ensure equal opportunity for inclusion in a curriculum. In addition, the initial list of clinical presentations may not have been all-inclusive, although we did strive to obtain saturation by sampling widely, including national and international guidelines and with local review by residents and attending physicians. Also, the results obtained with this tool are completely dependent on the key stakeholders being appropriately identified and adequately represented. Finally, our results may not be generalizable because the sample size was relatively small and the characteristics of our ICU rotation, including length and location (academic tertiary care), may not be similar to those in other centres. That being said, our goal was to describe a process for determining curricular content that could be used by others, rather than determining exactly what others should be teaching.

Future research should focus on establishing the validity of this tool, ideally by assessing learner and patient outcomes after a curriculum based on the content selected by the tool is implemented. In addition, exploration of the tool's characteristics, including the best titles for each of the scales, the ideal number of scales, establishing the construct validity of the scale labels and assessing a more detailed 5-point rating scale as compared to the current 3-point scale, would be important steps. Finally, in order to improve the generalizability and the validity of the content identified by the tool, the scope of future studies should be broadened to involve stakeholders from community ICUs, representatives from each of the training programmes that have their residents complete ICU rotations and potentially even patients.

## Conclusion

Curricular content is valid when it reflects patient case mix, the needs of learners and the expectations of teachers. Consequently, it may not be possible to create a single critical care curriculum that is valid for every ICU rotation. However, even if our content is not widely applicable, the process we used is, and that process can facilitate the creation of a reliable and valid curriculum.

## Key messages

• When it comes to teaching trainees in the ICU, there is a limited amount of time and a huge number of potential topics.

• Our tool allows for the most relevant topics to be selected easily, rapidly and reliably, and will provide content validity for any curriculum.

## Abbreviations

ICU: intensive care unit.

## Competing interests

The authors declare that they have no competing interests.

## Authors' contributions

ADP conceived of the study, participated in data collection and statistical analysis, and drafted the manuscript. KM helped to develop the study design, participated in statistical analysis and helped to draft the manuscript. JL helped to develop the study design and helped to draft the manuscript. TD helped to develop the study design, participated in statistical analysis and helped to draft the manuscript. All authors approved the final manuscript.
